# Retraction Notice: Optimized Turmeric Extract Reduces β-Amyloid and Phosphorylated Tau Protein Burden in Alzheimer's Transgenic Mice

**DOI:** 10.2174/1567205022999250430120355

**Published:** 2025-04-30

**Authors:** R. Douglas Shytle, Jun Tan, Paula C. Bickford, Kavon Rezaizadeh, L. Hou, Jin Zeng, Paul R. Sanberg, Cyndy D. Sanberg, Randall S. Alberte, Ryan C. Fink, Bill Roschek

**Affiliations:** a Center for Excellence in Aging and Brain Repair, Dept. of Neurosurgery;; b Silver Child Development Center, Dept. of Psychiatry and Behavioral Medicine,; c Neuroscience Program, University of South Florida College of Medicine, Tampa, *FL 33612;*; d Veterans Administration Hospital, Research Service, Tampa, FL 33612;; e Natura Therapeutics, Inc, 3802 Spectrum Blvd, Tampa, FL 33612;; f Deceased: October 4, 2010;; g Department of Food Science and Nutrition, University *of Minnesota, St. Paul, MN 55108; *; h HerbalScience Group LLC., Naples, FL 34110, USA

This article titled **“Retracted: Optimized Turmeric Extract Reduces β-Amyloid and Phosphorylated Tau Protein Burden in Alzheimer's Transgenic Mice,”** published in **Volume 9, Issue 4, 2012** of ***Current Alzheimer Research*** (10.2174/156720512800492459) has been retracted by the publisher following a thorough investigation that revealed potential data manipulation in the manuscript. As a result, the integrity and validity of the data presented could not be confirmed. The retraction has been made in agreement with the Editor-in-Chief. Despite multiple attempts, the authors did not respond to correspondence regarding this matter. Bentham Science apologizes to the readers of the journal for any inconvenience this may have caused. The Bentham Editorial Policy on Retraction can be found at https://benthamscience.com/editorial-policies-main.php

## BENTHAM SCIENCE DISCLAIMER:

It is a condition of publication that manuscripts submitted to this journal have not been published and will not be simultaneously submitted or published elsewhere. Furthermore, any data, illustration, structure or table that has been published elsewhere must be reported, and copyright permission for reproduction must be obtained. Plagiarism is strictly forbidden, and by submitting the article for publication the authors agree that the publishers have the legal right to take appropriate action against the authors, if plagiarism or fabricated information is discovered. By submitting a manuscript the authors agree that the copyright of their article is transferred to the publishers if and when the article is accepted for publication.

